# Effect of non-pharmacological interventions on the prevention of sarcopenia in menopausal women: a systematic review and meta-analysis of randomized controlled trials

**DOI:** 10.1186/s12905-023-02749-7

**Published:** 2023-11-14

**Authors:** Ting-Wan Tan, Han-Ling Tan, Min-Fang Hsu, Hsiao-Ling Huang, Yu-Chu Chung

**Affiliations:** 1 Department of Nursing, Hsinchu MacKay Memorial Hospital, Hsinchu, Taiwan; 2 Department of Orthopaedic Surgery, Faculty of Medicine, University of Malaya, Kuala Lumpur, Malaysia; 3 Department of Nursing, Yuanpei University of Medical Technology, Hsinchu, Taiwan; 4 Department of Healthcare Management, Yuanpei University of Medical Technology, Hsinchu, Taiwan

**Keywords:** Menopausal women, Exercise training, Vitamin D, Protein, Sarcopenia, Systematic review

## Abstract

**Background:**

Sarcopenia is a chronic disease marked by gradual muscle system and functional decline. Prior research indicates its prevalence in those under 60 varies from 8 to 36%. There is limited evidence on the effectiveness of non-pharmacological interventions for sarcopenia prevention in menopausal women aged 40–60. This study examines the influence of such interventions for sarcopenia prevention on these women.

**Methods:**

PubMed, EMBASE, Medline, Cochrane Library, CINAHL, PEDro, and Airiti Library were searched from inception until May 5, 2023. Randomized controlled trials that examined exercise, vitamin D and protein supplementation effects on muscle mass, strength, and physical function. Quality assessment used the Cochrane risk of bias tool, and analysis employed Comprehensive Meta-Analysis version 2.0.

**Results:**

A total of 27 randomized controlled trials, involving 1,989 participants were identified. Meta-analysis results showed exercise improved lean body mass (SMD = 0.232, 95% *CI*: 0.097, 0.366), handgrip strength (SMD = 0.901, 95% *CI*: 0.362, 1.441), knee extension strength (SMD = 0.698, 95% *CI*: 0.384, 1.013). Resistance training had a small effect on lean body mass, longer exercise duration (> 12 weeks) and higher frequency (60–90 min, 3 sessions/week) showed small to moderate effects on lean body mass. Vitamin D supplementation improved handgrip strength (SMD = 0.303, 95% *CI*: 0.130, 0.476), but not knee extension strength. There was insufficient data to assess the impact of protein supplementation on muscle strength.

**Conclusions:**

Exercise effectively improves muscle mass, and strength in menopausal women. Resistance training with 3 sessions per week, lasting 20–90 min for at least 6 weeks, is most effective. Vitamin D supplementation enhances small muscle group strength. Further trials are needed to assess the effects of vitamin D and protein supplementation on sarcopenia prevention.

**Registration number:**

This review was registered on PROSPERO CRD42022329273.

**Supplementary Information:**

The online version contains supplementary material available at 10.1186/s12905-023-02749-7.

## Background

The transition into menopause is associated with a decrease in the production and circulation of estrogen, which is a naturally occurring antioxidant in the body. This reduction leads to oxidative stress in various body tissues, including skeletal muscle, resulting in negative effects on muscle protein synthesis and mitochondrial dysfunction, these factors contribute to the loss of muscle mass and strength [2]. Sarcopenia is a chronic condition characterized by a progressive and degenerative loss of muscle mass, strength, and physical functional performance [1]. Menopause is a natural process that occurs in all women during their final menstrual period, typically between the ages of 40 and 60, and is characterized by a decrease in estrogen production and hormonal status [3]. On average, women experience a loss of approximately 0.6% of muscle mass in their 30s, and the rate of decline in muscle mass and muscle strength is even higher after the age of 50 [4]. Orprayoon et al. estimated the prevalence of pre-sarcopenia to be around 12% among menopausal women under the age of 65 [5]. Moreover, the presence of sarcopenia is associated with a negative impact on mental health, leading to a loss of self-confidence and social isolation, ultimately reducing the overall quality of life [6, 7]. Sarcopenia indirectly contributes to an increased risk of disability, reduced independence, and being bedridden, all of which are public health concerns and place a financial burden on healthcare and social care services [8]. Therefore, implementing health promotion and preventive interventions for sarcopenia is essential for public health.

The Asian and European Working Group for Sarcopenia supports non-pharmacological interventions such as exercise and nutrition as fundamental strategies. These interventions include exercise training, vitamin D supplementation, and protein supplementation to enhance motor neuron activity, maintain muscle elasticity, preserve muscle mass, rebuild muscle strength, and attenuate the progression of sarcopenia [1, 9].

Exercise training can enhance muscle activation, release inflammatory and hormonal substances, and activate skeletal muscle satellite cells [10, 11]. These mechanisms promote the muscle regeneration process, muscle protein synthesis, and help prevent muscle hypotrophy or muscle fiber hypoplasia [10, 11]. As a result, exercise training increases the cross-sectional area of muscles, improves muscle strength, and enhances physical functional performance [10, 11]. Vitamin D supplementation plays a crucial role in delaying skeletal muscle aging and increasing muscle protein synthesis. Vitamin D deficiency is associated with a risk of decreased muscle mass and strength, gait deficits, and an increased risk of falling [12]. Dietary protein intake is essential for enhancing muscle strength, muscle protein synthesis, and the growth factors involved in regulating skeletal muscle function and bone remodeling [13]. Therefore, non-pharmacological interventions are ideal goals to prevent or improve sarcopenia in menopausal women and should not be ignored.

Several systematic reviews and meta-analyses conducted between 2000 and 2023 have investigated non-pharmacological interventions to delay or prevent sarcopenia in post-menopausal women. Rubio-Arias et al. conducted a systematic review and meta-analysis based on data from five randomized controlled trials (RCTs) involving post-menopausal women aged 55 to 75 [14]. The studies included 8 to 32 weeks of whole-body vibration training and revealed no significant effect on lean body mass. Marín-Cascales et al. conducted a systematic review and meta-analysis based on data from 15 studies [15]. The analysis focused on post-menopausal women aged 65 and older who underwent 12 to 48 weeks of multi-component exercise training. The findings indicated beneficial effects in increasing or preventing the loss of muscle mass, although high heterogeneity among the studies was noted. Thomas et al. conducted a systematic review and meta-analysis based on data from 26 studies that included post-menopausal women aged 50 to 80 [16]. The analysis focused on the effects of 16 weeks of resistance exercise training on lean body mass, and it showed enhancements in lean body mass. Tabrizi et al. reported a meta-analysis based on 12 studies involving post-menopausal women aged 45 to 86 [17]. The studies investigated the effects of daily intake ranging from 10 international units (IU) to 50,000 IU of vitamin D supplementation for 12 to 96 weeks. The findings showed no significant effect on the timed up and go test, which assesses mobility. Abshirini et al. conducted a meta-analysis based on 29 RCTs involving post-menopausal women aged 65 and older. The studies examined the effects of daily or weekly intake of vitamin D2 or vitamin D3 supplementation ranging from 1,000 IU to 100,000 IU for 1 to 60 months [18]. The analysis showed no beneficial effect on handgrip strength and the timed up and go test. Zhang et al. conducted a meta-analysis based on 13 RCTs involving post-menopausal women aged 45 to 99 [19]. The studies evaluated the effects of daily or weekly intake of vitamin D2 or vitamin D3 supplementation ranging from 1,000 IU to 100,000 IU for 3 to 60 months. The findings showed a beneficial effect on handgrip strength but no significant effect on the timed up and go test.

These systematic reviews and meta-analyses provide insights into the effectiveness of non-pharmacological interventions and vitamin D supplementation in post-menopausal women with sarcopenia. Previous meta-analyses on exercise regimes for postmenopausal women have had methodological issues, resulting in uncertain protocols. Both vitamin D and protein seem to offer benefits for muscle health in postmenopausal older women, yet the effects of menopause on muscle changes have not been comprehensively investigated. Middle-aged women, undergoing menopausal transition, have exhibited variations in muscle morphology. Nevertheless, no prior review has thoroughly examined non-pharmacological interventions for middle-aged women. Thus, this systematic review and meta-analysis aims to analyze the influence of exercise, vitamin D, and protein on sarcopenia, specifically in menopausal women aged 40 to 60 needs to be explored and seems warranted. The study will employ a systematic evaluation of data from selected RCTs, by addressing this knowledge gap, the study aims to provide valuable information that can be applied in clinical contexts and contribute to achieving public health goals.

## Methods

### Design

This systematic review and meta-analysis of randomized controlled trials was registered at PROSPERO, protocol was not published previously. The reporting of this review was followed by the Preferred Reporting Items for Systematic Reviews and Meta-Analyses (PRISMA) statement [20] Supplementary Material 1.

### Data sources and search strategies

The study conducted searches in the following electronic database for articles published from 1989 (sarcopenia was first proposed by Rosenberg in 1989) to May 5, 2023; databases searched were PubMed, EMBASE, Medline, Cochrane Library, CINAHL, PEDro and Airiti Library. Manual search was conducted to enhance accuracy and completeness. In accordance with the PICOS (Population, Intervention, Comparison, and Outcome) as the search guide approach. Search keywords were selected from the medical subject heading terms (MeSH), searched terms were those related to midlife menopause women (“midlife women” or “middle aged women” AND “perimenopause” or “menopausal transition” or “climacteric”) combined with terms related to exercise, vitamin D and protein non-pharmacological interventions (“exercise” or “fitness training” or “vitamin d” or “protein*” or “soy products”) and terms related to sarcopenia (“sarcopenia” or “lean body mass” or “muscle mass” or “muscle strength” or “handgrip” or “leg extension” or “physical performance” or “physical functional performance”) and study type (“randomized controlled trial” or “randomized controlled study” or “randomization”). The final searches were conducted with appropriate specifications for each database, following the PICOS format.

### Eligibility criteria

This systematic review focuses on peer-reviewed literature that meets the following eligibility criteria. Population (P): The study includes menopausal women between the ages of 40 and 60. Intervention (I): The review considers exercise training interventions without any restrictions on the modalities, duration, frequency, or intensities. Additionally, investigations of the effect of vitamin D and protein supplementation are included, without limitations on the forms, doses, or durations of supplementation. Comparison (C): Results are compared with a control group, which can be either an active control (such as placebo control) or a passive control (such as usual care or no intervention in exercise, vitamin D supplementation, or protein supplementation). Outcome (O): The review examines measures of muscle mass (lean body mass), muscle strength (handgrip strength and knee extension strength), and physical functional performance (timed up and go test). Lean body mass is assessed using dual-energy X-ray absorptiometry, bioelectrical impedance analysis, computed tomography, or magnetic resonance imaging. Muscle strength is measured using an isokinetic dynamometer or grip dynamometer [1, 9]. Physical functional performance is evaluated using the timed up and go test [21]. The study design includes RCTs published in English or Chinese. The following are non-inclusion criteria: (1) women with therapeutically induced menopause, (2) women with cancer or severe acute or chronic illnesses, (3) women undergoing hormonal therapy or medical treatments such as chemotherapy, surgery, or psychiatric treatment, (4) non RCTs, cohort studies, and meta-analysis studies. Studies published only as abstracts are also excluded from the review.

### Study selection

Two independent reviewers conducted an initial screening of the titles and abstracts of the studies based on the predefined inclusion and exclusion criteria. The full text of articles that met the inclusion criteria was then retrieved. All potentially eligible studies were independently evaluated by the two reviewers, again using the predefined inclusion criteria. In case of any disagreements or discrepancies between the reviewers, a consensus was reached through discussion and mutual agreement.

### Data collection and extraction

The data collection and extraction process were conducted using EndNote X9 (Clarivate Analytics, Philadelphia, PA, USA). Microsoft Word 2007 was utilized for the coding procedure of the data items from the included studies. Two independent reviewers were responsible for coding and auditing the necessary data, and the results were entered separately.

For each included randomized controlled trial, the researchers collected the following information: General study information, including the first author’s name and year of publication. Additionally, characteristics of the participants, such as sample size and attrition rate, were recorded. Intervention details were carefully documented. This included information on the type of intervention (e.g., exercise, vitamin D, protein) as well as specific characteristics of the intervention, such as the dose, frequency, intensity, and duration. Detailed information on the outcomes measured in each study was also collected.

### Risk of bias assessment

To assess the quality of the included studies, the risk of bias was evaluated using the Cochrane Collaboration’s tool for randomized trials, known as Risk of Bias 2 (RoB 2). This tool, as described in the Cochrane Handbook for Systematic Reviews of Interventions [22], consists of five domains that were assessed for individually RCTs. The first domain focuses on bias arising from the randomization process. It examines whether the randomization was performed appropriately and whether any imbalances occurred between the intervention and control groups. The second domain assesses bias due to deviations from intended interventions. This domain examines if there were any differences in the implementation of the interventions or if participants received additional treatments that may have influenced the outcomes. The third domain examines bias due to missing outcome data. It considers whether there were any missing data and whether the missingness could have introduced bias in the results. The fourth domain addresses bias in the measurement of the outcome. It evaluates the methods used to measure the outcomes and determines whether they were reliable and valid. The fifth domain focuses on bias in the selection of the reported result. It examines whether there was selective reporting of outcomes or analysis methods that could have influenced the findings. For each domain, specific questions were considered, and judgments were made based on the available information. The judgments were categorized as “low risk of bias,“ “some concerns,“ or “high risk of bias” to provide an overall assessment of the risk of bias for each included study.

### Grading the certainty of evidence

The certainty of evidence regarding outcomes was assessed according to the Grading of Recommendations, Assessment, Development, and Evaluation (GRADE) handbook [23]. The GRADE approach was employed to evaluate evidence certainty based on five criteria: risk of bias, inconsistency, indirectness, imprecision, and publication bias. Certainty ratings were categorized into four levels of the system: high, moderate, low, or very low [23].

### Data synthesis

Statistical analysis was performed using Comprehensive Meta-Analysis version 2.0 [24]. The risk of bias was assessed using the RoB 2 standard template [22]. To calculate the overall effect size of the intervention and minimize deviations between measurement variables, the standardized mean difference (SMD) with 95% confidence intervals (95% *CI*) was utilized for continuous data. Statistical heterogeneity was evaluated using Cochrane’s Q statistic, which compares the effect sizes of each study [25]. The *I*^*2*^ value was used to assess the degree of inconsistency across studies in the meta-analysis. The *I*^*2*^ values were categorized as follows: very low (0–25%), low (25–50%), medium (50–75%), and high (> 75%), indicating the level of heterogeneity [26]. A fixed-effect model was employed for data pooling. However, if significant heterogeneity was observed (*p* < .10 and *I*^*2*^ > 50%), a random-effects model was applied [27]. The effect sizes of the SMD were interpreted based on Cohen’s interpretation of effect size. Effect sizes of 0.2, 0.5, and 0.8 were considered small, medium, and large effects, respectively [28]. Sensitivity analyses and subgroup analyses were conducted to assess the impact of each included study on the pooled SMD and investigate potential sources of heterogeneity [29]. Publication bias was evaluated using funnel plots, and Egger’s weighted regression tests were visually inspected [30].

## Results

### Study search outcomes

**Fig. 1:**
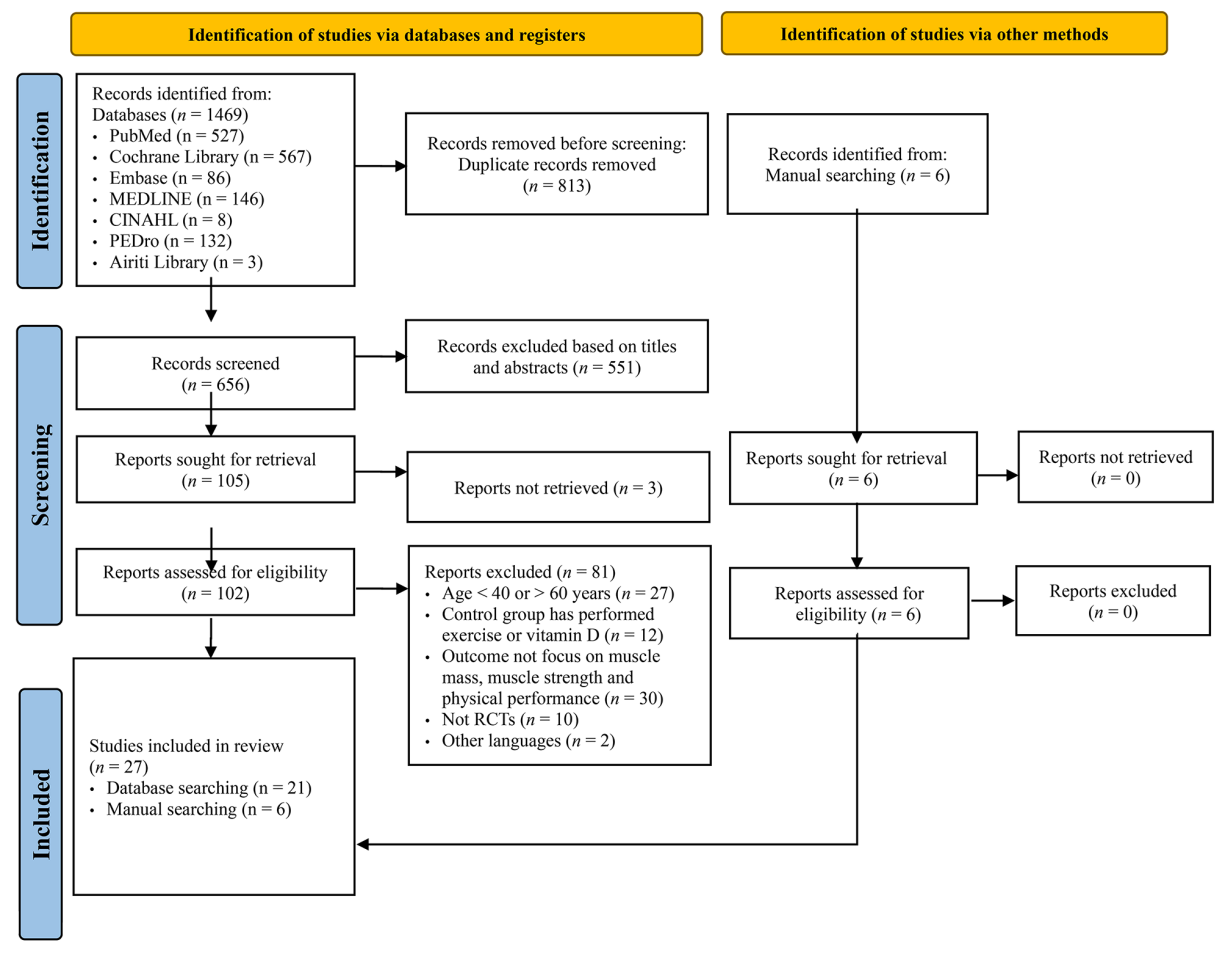
PRISMA flow diagram for selection process of included studies

Figure 1 presents the PRISMA 2020 flowchart illustrating the study inclusion process. A total of 1,469 studies were identified through searches conducted in 7 electronic databases. Using EndNote X9 (Clarivate Analytics, Philadelphia, PA, USA), 813 duplicate articles were removed, leaving 656 unique articles. Following the analysis of titles and abstracts, 551 articles were excluded as they did not meet the inclusion criteria. Full texts were not available for 3 articles. The eligible full texts of 102 articles were further assessed for eligibility. Through manual retrieval, 6 additional articles were included. In total, 27 studies [31–57] were included in the meta-analysis: 18 studies focused on exercise training, 5 studies examined the effects of vitamin D, and 4 studies investigated the impact of protein supplementation (one study overlapped with exercise training).

### Quality appraisal of included studies

**Fig. 2:**
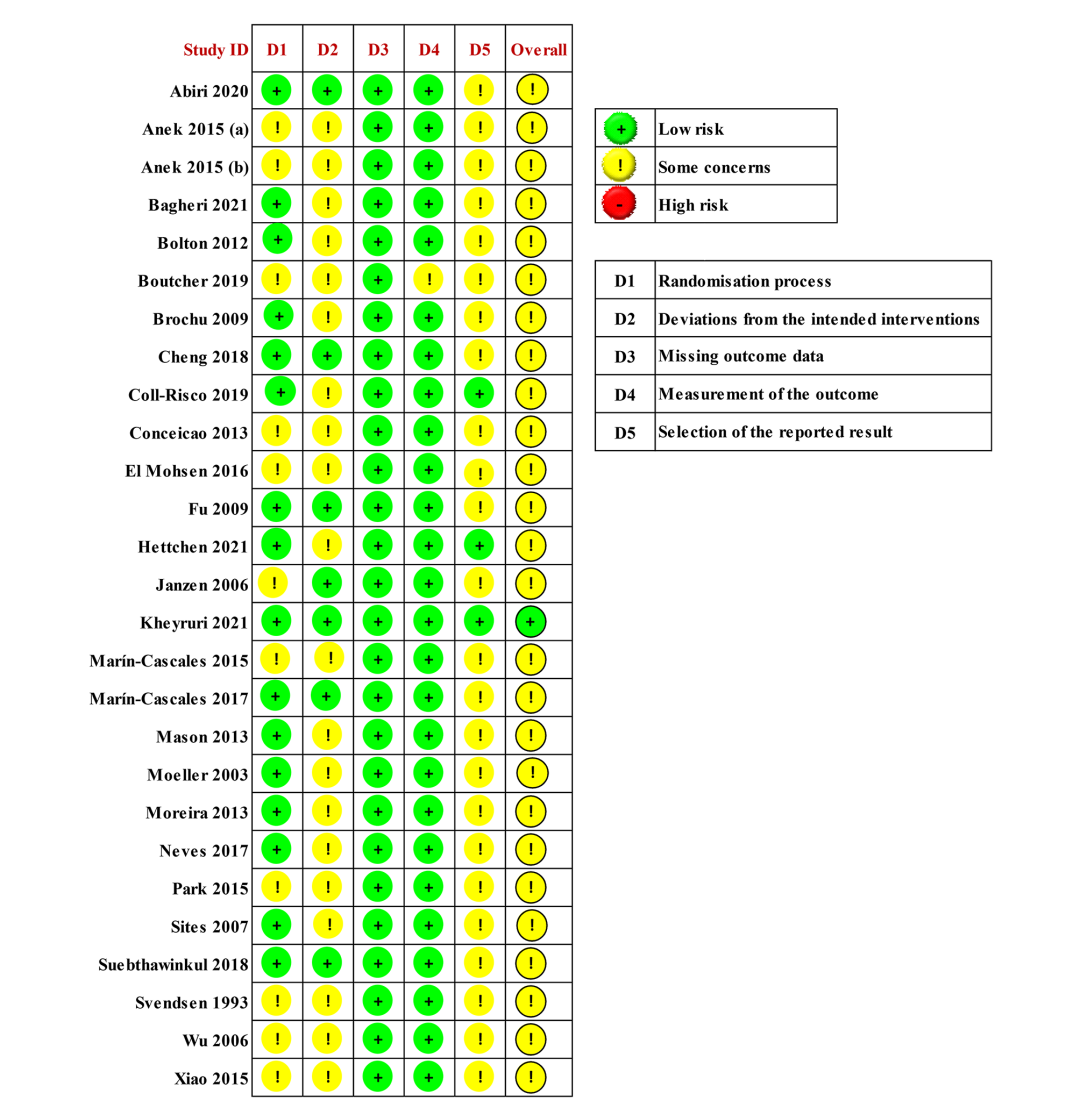
Risk of bias summary of individual studies

Figure 2 presents the results of the risk of bias assessment using RoB 2.0 for the 27 included studies [31–57]. Among the 27 studies, 26 were judged to have some concerns regarding bias, while one study was assessed as having a low risk of bias. The judgments of some concern bias were primarily due to the absence of specific information regarding the generation of the randomization sequence, lack of clarity regarding blinding procedures, and insufficient information about the randomization allocation process. Moreover, data from the studies did not indicate the presence of a pre-specified plan or clinical trial registration, which affected the analysis. Overall, the risk of bias judgments for the included studies ranged from low risk to some concerns. Two independent reviewers assigned scores to assess the consistency level of each domain, achieving a score of 96.4% agreement.

### GRADE analysis

The outcomes of exercise training on lean body mass and knee extension strength, as well as the outcome of vitamin D on handgrip strength, received a moderate level of certainty of evidence. However, the effectiveness of exercise on handgrip strength was rated as having a low level of certainty. This downgrade was attributed to uncertainties in the estimate of the effect, suggesting that the true effect might differ substantially from the estimated effect. The outcomes of both vitamin D on handgrip strength and protein on lean body mass garnered a low level of certainty of evidence. This downgrade resulted from a significant risk of bias, arising from inadequate reporting on methods of allocation concealment, and notable imprecision, as evidenced by the confidence interval crossing the line of no effect (Table 1).

**Table 1:** GRADE analysis of the overall measurement outcomes **Exercise compared to no exercise in preventing sarcopenia**

Exercise compared to no exercise in preventing sarcopenia											
Certainty assessment							No of patients		Effect		Certainty
No of studies	Study design	Risk of bias	Inconsistency	Indirectness	Imprecision	Other considerations	Exercise	No exercise	Relative (95% CI)	Absolute (95% CI)	
**Lean Body Mass**											
14	RCTs	serious^a^	not serious	not serious	not serious	none	570	483	-	SMD 0.232 (0.097 to 0.366)	⨁⨁⨁◯ Moderate
**Handgrip Strength**											
2	RCTs	serious^a^	not serious	not serious	serious^b^	none	30	30	-	SMD 0.901 (0.362 to 1.411)	⨁⨁◯◯ Low
**Knee Extension Strength**											
6	RCTs	serious^a^	not serious	not serious	not serious	none	160	90	-	SMD 0.698 (0.384 to 1.013)	⨁⨁⨁◯ Moderate
**Vitamin D compared to no vitamin D in preventing sarcopenia**											
**Certainty assessment**							**No of patients**		**Effect**		**Certainty**
**No of studies**	**Study design**	**Risk of bias**	**Inconsistency**	**Indirectness**	**Imprecision**	**Other considerations**	**Vitamin D**	**No vitamin D**	**Relative** **(95% CI)**	**Absolute** **(95% CI)**	
**Handgrip Strength**											
4	RCTs	serious^a^	not serious	not serious	not serious	none	212	213	-	SMD 0.267 (0.065 to 0.469)	⨁⨁⨁◯ Moderate
**Knee Extension Strength**											
3	RCTs	serious^a^	not serious	not serious	Serious^c^	none	108	108	-	SMD 0.269 (0.006 to 0.545)	⨁⨁◯◯ Low
**Protein compared to no protein in preventing sarcopenia**											
**Certainty assessment**							**No of patients**		**Effect**		**Certainty**
**No of studies**	**Study design**	**Risk of bias**	**Inconsistency**	**Indirectness**	**Imprecision**	**Other considerations**	**Protein**	**No protein**	**Relative** **(95% CI)**	**Absolute** **(95% CI)**	
**Lean body mass**											
3	RCTs	serious^a^	not serious	not serious	Serious^c^	none	91	74	-	SMD 0.074 (0.267 to 0.414)	⨁⨁◯◯ Low

RCTs: randomized controlled trials; CI: confidence interval; SMD: standardised mean difference

Explanations:

a. Downgraded one level because of overall unclear risk of bias

b. Downgraded one level because of widely differing estimates of the treatment effect

c. Downgraded one levels because the 95% confidence interval includes no effect

### Study characteristics

Table 2 provides a summary of the characteristics of the included studies. Among the 27 studies [31–57], three were conducted in Thailand, Australia, Canada, Spain, and Brazil, respectively (55.5%), while two studies were conducted in the United States, China, and Iran, respectively (22.2%). Additionally, one study was conducted in Finland, Korea, Italy, Denmark, Japan, and Egypt, respectively. Among the included studies, a total of 18 focused on exercise training. These studies were involved a total of 1,327 participants. The exercise training modalities varied, with 10 studies focusing on resistance exercise training (including resistance training, aquatic exercise, weight-bearing exercise, balance training, functional training, bilateral and unilateral strength training), five studies on aerobic exercise training (including circuit aerobic step exercise, sprint interval training, multi-component training, tai chi ball exercise, and walking exercise), and four studies on other forms of exercise training (including whole-body vibration and combined exercise). For vitamin D supplementation, a total of five studies were included. These studies were involved 477 participants. Different forms of vitamin D were used, including vitamin D, vitamin D2, and vitamin D3. Regarding protein supplementation, five studies were included, one of which was also included in the exercise training category. These studies were involved 185 participants. Various forms of protein were used, including soy protein, branched-chain amino acids, and isoflavones.

**Table 2:** Characteristics of included studies

First author/year (Country)	Average age (Mean ± SD) / Health condition	Attrition rate (%)/Sample size (*n*)	Intervention type	Intervention (dose/frequency/intensity)	Intervention duration	Outcomes measured
Abiri 2020 (Iran) [31]	EG = 45.2 ± 2.6 CG = 45.7 ± 3.1 Healthy	4.05% EG = 37 CG = 37	vitamin D	• 1000IU/day	12 weeks	• handgrip strength (kg) • knee extension strength (kg) • timed up and go test (s)
Anek 2015 (a) (Thailand) [32]	EG = 51.0 ± 3.0 CG = 50.7 ± 3.1 Healthy	0% EG = 26 CG = 26	vitamin D_2_	• 5000IU/week	4 weeks	• knee extension (kg/bw)
Anek 2015 (b) (Thailand) [33]	EG = 50.7 ± 3.0 CG = 50.9 ± 3.3 Healthy	0% EG = 26 CG = 26	circuit aerobic step exercise	• 50 min/time, 3 sessions/week • 55–75% HRmax	4 weeks	• knee extension (kg/bw)
Bagheri 2021 (Canada) [34]	56 ± 3.7 Healthy	0% EG = 10 CG = 10	branched-chain amino acid	• 9 g/day	8 weeks	• muscle mass (kg) • handgrip strength (kg)
Bolton 2012 (Australia) [35]	EG = 60.3 ± 5.6 CG = 56.3 ± 4.7 Hip osteopenia	5.13% EG = 19 CG = 20	resistance training exercise	• 60 min/time, 3 sessions/week • 8–12 repetitions	52 weeks	• lean body mass (g) • knee extensors (Nm/kg)
Boutcher 2019 (Australia) [36]	EG = 54.1 ± 3.6 CG = 53.3 ± 3.4 Healthy	0% EG = 20 CG = 20	sprint interval training	• 20 min/time, 3 sessions/week • 80–85% HRpeak	8 weeks	• lean body mass (kg)
Brochu 2009 (Canada) [37]	EG = 57.2 ± 5.0 CG = 58.0 ± 4.7 Overweight	21.9% EG = 48 CG = 89	resistance training + caloric restriction	• 60 min/time, 3 sessions/week • 75–80% HRmax, 12 repetition	24 weeks	• lean body mass (kg)
Cheng 2018 (China) [38]	EG = 58.2 ± 8.1 CG = 57.1 ± 8.2 Healthy	18.5% EG = 86 CG = 87	vitamin D_3_	• 0.5 µg/day	12 weeks	• handgrip strength (kg)
Coll-Risco 2019 (Spain) [39]	EG = 52.8 ± 4.5 CG = 52.7 ± 4.5 Healthy	24% EG = 75 CG = 75	concurrent exercise program (aerobic + resistance training)	• 60 min/time, 3 sessions/week • 12–16 Borg perceived exertion	16 weeks	• lean body mass (kg)
Conceição 2013 (Brazil) [40]	EG = 53.4 ± 3.9 CG = 53.0 ± 5.7 Healthy	0% EG = 10 CG = 10	resistance training	• 3 sessions/week • 50–70% 1-RM, 8–10 repetition	16 weeks	• lean body mass (kg)
El Mohsen 2016 (Egypt) [41]	EG = 59.1 ± 4.1 CG = 58.6 ± 3.9 Osteopenia	0% EG = 12 CG = 12	weight-bearing exercise	• 5 sessions/week • 15–20 repetitions	6 weeks	• knee extensors (Nm/kg)
Fu 2009 (Australia) [42]	EG = 52.2 ± 5.6 CG = 51.3 ± 5.4 Healthy	30% EG = 26 CG = 24	balance training	• 60 min/time, 2 sessions/week • Not applicable	12 weeks	• timed up and go test (s)
Hettchen 2021 (Finland) [43]	EG = 53.6 ± 2.0 CG = 54.5 ± 1.6 Osteopenia or osteoporosis	24% EG = 27 CG = 27	resistance training	• 40–60 min/time, 3 sessions/week • 80–85% HRmax, 8-16repetition	52 weeks	• lean body mass (kg)
Janzen 2006 (Canada) [44]	EG1 = 55.8 ± 8.2 EG2 = 54.8 ± 6.5 CG = 58.8 ± 6.7 Healthy	12.3% EG1 = 15 EG2 = 14 CG = 28	• EG1: bilateral strength • EG2: unilateral strength	• 3 sessions/week • 50–60% 1-RM, 12 repetition	26 weeks	• lean body mass (kg) • knee extension (kg)
Kheyruri 2021 (Iran) [45]	EG = 45.0 ± 1.3 CG = 46.8 ± 1.5 Vitamin D deficiency	7.8% EG = 45 CG = 45	vitamin D	• 50,000IU/week	8 weeks	• handgrip strength (kg) • knee extension strength (kg) • timed up and go test (s)
Marín-Cascales 2015 (Spain) [46]	60.0 ± 6.3 Healthy	42.2% EG1 = 25 EG2 = 25 CG = 15	• EG1: whole-body vibration • EG2: multicomponent training	• 60 min/time, 3 sessions/week • EG1: 35 Hz • EG2: 50–60% HRmax	12 weeks	• lean body mass (kg) • knee extension (Nm/kg)
Marín-Cascales 2017 (Spain) [47]	60.0 ± 6.3 Healthy	40.6% EG1 = 25 EG2 = 25 CG = 15	• EG1: whole-body vibration • EG2: multicomponent training	• 60 min/time, 3 sessions/week • EG1: 35–40 Hz • EG2: 50–75% HRmax	24 weeks	• lean body mass (kg) • knee extension (Nm/kg)
Mason 2013 (USA) [48]	57.9 ± 5.0 Overweight or obese	9.11% EG1 = 118 EG2 = 117 EG3 = 117 CG = 87	• EG1: diet control • EG2: aerobic exercise • EG3: diet control + aerobic exercise	• 45 min/time, 5 sessions/week • 70–85% HRmax	48 weeks	• lean body mass (kg)
Moeller 2003 (USA) [49]	50.6 (median age) Healthy	12.7% EG1 = 24 EG2 = 24 CG = 31	• EG1: aglycone (soy protein) • EG2: aglycone (soy protein)	• EG1: 80.4 mg (40 g)/day • EG2: 4.4 mg (20 g)/day	24 weeks	• lean body mass (kg)
Moreira 2013 (Brazil) [50]	EG = 58.6 ± 6.7 CG = 59.3 ± 6.1 Healthy	7.41% EG = 64 CG = 44	aquatic exercise	• 60 min/time, 3 sessions/week • 55–60% HRmax	24 weeks	• knee extensor (kg) • handgrip strength (kg) • timed up and go test (s)
Neves 2017 (Brazil) [51]	EG = 58.6 ± 3.9 CG = 57.7 ± 4.8 Healthy	15.6% EG = 32 CG = 32	functional training	• 30 min/time, 3 sessions/week • 3 sets of 70 s, 30 s interval	16 weeks	• lean body mass (kg)
Park 2015 (Korea) [52]	EG = 57.2 ± 2.6 CG = 57.2 ± 1.7 Obesity	0% EG = 10 CG = 10	combined exercise (resistance equipment training + treadmill acclimation exercises)	• 40 min/time, 3 sessions/week • 60–75% 1RM, 8-15repetitions	12 weeks	• lean body mass (kg) • handgrip strength (kg)
Sites 2007 (Italy) [53]	EG = 55.0 ± 5.4 CG = 57.8 ± 4.3 Healthy	16.7% EG = 9 CG = 9	soy protein + isoflavones	• 20 g + 160 mg	12 weeks	• lean body mass (kg)
Suebthawinkul 2018 (Thailand) [54]	EG = 55.1 ± 2.9 CG = 54.9 ± 3.4 Healthy	1.1% EG = 44 CG = 44	vitamin D_2_	• 40,000IU/week	12 weeks	• muscle mass (kg) • handgrip strength (kg)
Svendsen 1993 (Demark) [55]	53.8 ± 2.5 Healthy	2.48% EG1 = 51 EG2 = 49 CG = 21	• EG1: diet • EG2: diet + exercise (aerobic exercise + RT)	• 60–90 min/time, 3 sessions/week • 70% HRmax, 7-15repetitions	12 weeks	• lean body mass (kg)
Wu 2006 (Japan) [56]	EG1 = 55.2 ± 2.8 EG2 = 53.8 ± 2.9 EG3 = 54.4 ± 2.9 CG = 54.9 ± 2.9 Healthy	5.9% EG1 = 34 EG2 = 34 EG3 = 34 CG = 34	• EG1: walking • EG2: 75 mg isoflavones • EG3: 75 mg isoflavones + walking	• 60 min/time, 3 sessions/week • 5 to 6 km/h	24 weeks	• lean body mass (kg)
Xiao 2015 (China) [57]	55.5 Healthy	0% EG = 20 CG = 20	tai chi ball exercise	• 60–120 min/time, 3 sessions/week	24 weeks	• handgrip strength (N)

*Note*. EG: experimental group; CG: control group

### Meta-analytic synthesis of results

For the timed up and go test, two studies examined the effect of exercise training [41, 49], while two studies investigated the effect of vitamin D supplementation [30, 44]. Regarding muscle mass, two studies reported the effect of vitamin D supplementation [37, 53]. One study assessed the effect of protein supplementation on handgrip strength [33]. However, no studies provided data on the effect of protein supplementation on physical functional performance, thus preventing a meta-analysis in this regard due to insufficient data.

### Effects of exercise training on measures of lean body mass

**Fig. 3:**
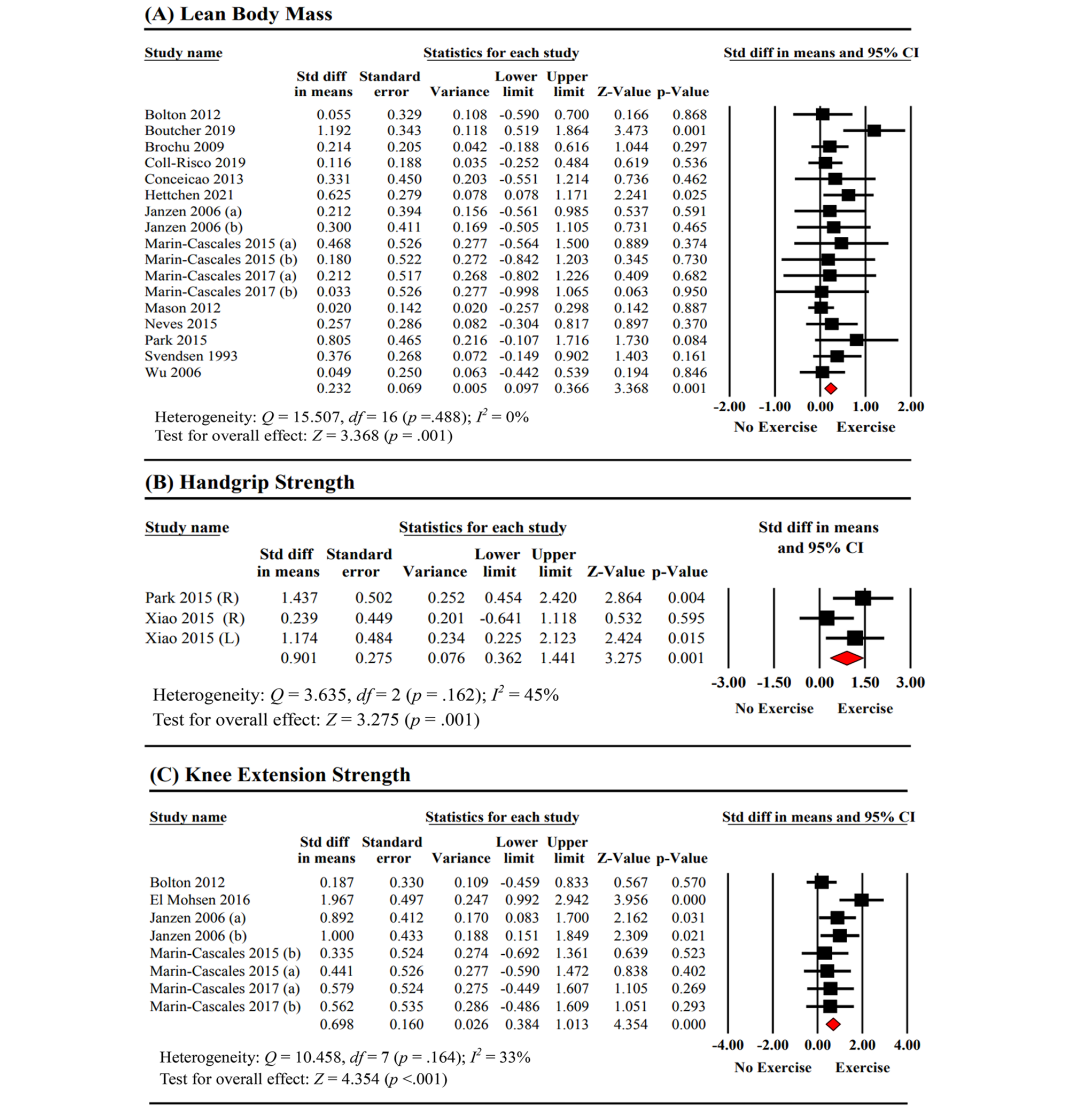
Meta-analysis of the effects of exercise training on lean body mass, handgrip strength, and knee extension strength between the two groups

Fourteen studies (17 datasets) [35–37, 39, 40, 43, 44, 46–48, 51, 52, 55, 56] with a total of 1,053 participants were included in the meta-analysis to assess the effects of exercise training on lean body mass. The meta-analysis showed homogeneity among the studies (*Q* = 15.507, *p* = .488, *I*^2^ = 0%) (Fig. 3-A, Table 3). The pooled analysis of the data demonstrated a significant overall improvement in lean body mass due to exercise training (SMD = 0.232, 95% CI: 0.097, 0.366, *p* = .001). The funnel plot did not indicate obvious asymmetry, and Egger’s linear regression test did not show evidence of publication bias in the meta-analysis of exercise training on lean body mass (*p* = .073). Subgroup analyses were conducted based on exercise type (resistance exercise, aerobic exercise, and other exercise), exercise duration (≤ 12 weeks and > 12 weeks), and exercise frequency (20–45 min three sessions per week and 60–90 min three sessions per week). Detailed results can be found in Table 2.

**Table 3:** Effect sizes of the overall and subgroup analysis

Title	Studies (*n*)	Participants (*n*)	Effect size SMD (95% *CI*)	*p* value	Heterogeneity *I*^*2*^ value
Overall effect by exercise training	14				
Lean body mass	14	1053	0.232 (0.097, 0.366)	0.001	0%
Handgrip strength	2	60	0.901 (0.362, 1.441)	0.001	45%
Knee extension strength	5	250	0.698 (0.384, 1.013)	< 0.001	33%
Subgroup analysis by types of exercise training					
Resistance exercise	7	391	0.316 (0.097, 0.535)	0.005	0%
Aerobic exercise	4	378	0.155 (-0.062, 0.372)	0.160	61%
Others	3	284	0.222 (-0.056, 0.500)	0.118	0%
Subgroup analysis by duration of exercise training					
≤ 12 weeks	4	195	0.581 (0.247, 0.916)	0.001	28%
> 12 weeks	10	858	0.164 (0.016, 0.311)	0.029	0%
Subgroup analysis by frequency of exercise training					
20–45 min/3 sessions/week	4	171	0.593 (0.278, 0.909)	< 0.001	17%
60–90 min/3 sessions/week	10	882	0.151 (0.002, 0.300)	0.048	0%
Overall effect by vitamin D	4				
Handgrip strength	4	425	0.267 (0.065, 0.469)	0.009	0%
Knee extension strength	3	216	0.269 (-0.006, 0.545)	0.055	17%
Overall effect by protein	3				
Lean body mass	3	165	0.074 (-0.267, 0.414)	0.671	0%

*Note*. SMD: standard mean difference; *CI*: confidence intervals; statistical method: fixed effect model

#### Effects of exercise training on measures of handgrip strength

Two studies [52, 57] (three datasets) with a total of 60 participants investigated the effects of exercise training on handgrip strength. The meta-analysis showed a significant but relatively low heterogeneity among the studies (*Q* = 3.635, *p* = .162, *I*^2^ = 45%). The overall pooled results indicated a significant improvement in handgrip strength due to exercise training (SMD = 0.901, 95% CI: 0.362, 1.441, *p* = .001) (Fig. 3-B, Table 3). Egger’s linear regression test did not reveal any indication of publication bias in the meta-analysis of exercise training on handgrip strength (*p* = .073).

#### Effects of exercise training on measures of knee extension strength

Six studies [31, 35, 41, 44, 46, 47] (nine datasets) with a total of 250 participants investigated the effects of exercise training on knee extension strength. The meta-analysis initially showed a significant high heterogeneity among the studies (Q = 34.114, *p* < .001, *I*^2^ = 77%). However, sensitivity analyses were performed by removing a study [31] with inadequate or unclear data, and the results remained consistent, indicating the reliability and robustness of the findings. After the sensitivity analyses, the meta-analysis demonstrated a significant but relatively low heterogeneity among the studies (*I*^2^ = 33%). Based on five studies [35, 41, 44, 46, 47] (eight datasets) with 187 participants, the overall pooled results showed a significant improvement in knee extension strength due to exercise training (SMD = 0.698, 95% *CI*: 0.384, 1.013, *p* < .001) (Fig. 3-C, Table 3). Egger’s linear regression test did not reveal any indication of publication bias in the meta-analysis of exercise training on knee extension strength (*p* = .446).

#### Effects of vitamin D on measures of handgrip strength

The meta-analysis of handgrip strength included 4 studies [31, 38, 45, 54] with 425 participants supplemented with vitamin D. The meta-analysis manifested a significant very low heterogeneity among the studies (*Q* = 0.422, *p* = .936, *I*^2^ = 0%). Overall pooled results showed a significant improved in handgrip strength (SMD = 0.267, 95% *CI*: 0.065, 0.469, *p* = .009) (Table 3). Egger’s linear regression test revealed no indication of publication bias in the meta-analysis of vitamin D supplementation on handgrip strength (*p* = .958).

#### Effects of vitamin D on measures of knee extension strength

The meta-analysis of knee extension strength, based on 3 studies [31, 32, 45] involving 216 participants who received vitamin D supplementation, demonstrated a significant and very low heterogeneity among the studies (*Q* = 2.407, *p* = .300, *I*^2^ = 17%). The overall pooled results showed no significant effect on knee extension strength with vitamin D supplementation (Table 3). Egger’s linear regression test indicated no indication of publication bias in the meta-analysis of vitamin D supplementation on knee extension strength (*p* = .247).

#### Effects of protein on measures of lean body mass

The meta-analysis of lean body mass included 3 studies [49, 53, 56] (4 datasets) with 165 participants supplemented with protein. The meta-analysis manifested a significant very low heterogeneity among the studies (*Q* = 0.426, *p* = .935, *I*^2^ = 0%). Overall pooled results with no significant effect were observed in lean body mass (Table 3). Egger’s linear regression test revealed no indication of publication bias in the meta-analysis of protein supplementation on lean body mass (*p* = .198).

## Discussion

This study is pioneering in its determination of sarcopenia indicators based on the guidelines from both the Asian and European working groups [1, 9]. Additionally, the study offers new insights by conducting a comprehensive meta-analysis to evaluate the effects of non-pharmacological interventions on preventing sarcopenia among menopausal women aged 40 to 60.

The results of our meta-analysis revealed a small effect of exercise training on lean body mass, which is consistent with the findings of a previous review conducted by Marín-Cascales et al. [15] and Benton et al.‘s study [58]. However, it is important to note that our review and Benton et al.‘s study [58] focused on a narrower age group (40 to 60 years old) compared to the broader age range (48 to 95 years old) examined in Marín-Cascales et al.‘s review. Despite this difference, both studies suggest that exercise training has a beneficial impact on lean body mass across different age groups. The underlying mechanisms responsible for the observed effects of exercise training on lean body mass are multifactorial. One proposed mechanism involves the reduction of inflammatory-related cytokines and the induction of growth hormone responses [59]. These factors contribute to muscle repair and regeneration, as well as improvements in mitochondrial function within muscle cells, ultimately leading to the formation of new skeletal muscle tissue. These findings highlight the importance of exercise training in mitigating the reduction in muscle cross-sectional area and the loss of muscle mass associated with aging [11]. Overall, our meta-analysis supports the notion that exercise training is beneficial for increasing lean body mass, particularly in the age range of 40 to 60 years old. Further research is needed to explore the specific exercise modalities, durations, and intensities that yield the most substantial gains in lean body mass in this population. Additionally, future studies should investigate the long-term effects of exercise training on lean body mass and its implications for overall health and functional outcomes.

A subgroup analysis of exercise type revealed that resistance exercise had a small positive effect on lean body mass. This finding aligns with a previous systematic review and meta-analysis conducted by Thomas et al. [16]. Their study, which included 26 studies, demonstrated a mean increase of 0.43 kg in lean body mass after 16 weeks of resistance training in postmenopausal women aged between 50 and 80 years. Furthermore, Benton et al.‘s non-randomized controlled study revealed an increase of 1.1 ± 0.3 kg in lean body mass after completing 8 weeks of progressive resistance training among untrained middle-aged women aged 40 to 55 [58]. Resistance training has been shown to increase the secretion of growth hormone, which stimulates skeletal muscle protein synthesis and helps prevent muscle mass loss [60]. In contrast, our subgroup analysis indicated that aerobic exercise alone did not have a significant positive effect on lean body mass, which is consistent with a prior study by Willis et al. [61]. In their RCTs, they observed a decrease in lean body mass (-0.10 kg) following 8 months of aerobic exercise. In contrast, resistance exercise (+ 1.09 kg) and multi-component exercise (+ 0.81 kg) resulted in positive effects on lean body mass over the same duration. The reason for the negative effect of aerobic exercise on lean body mass is attributed to the fact that different types of exercise elicit distinct physiological responses in muscle cell and tissue contractile function [62]. While aerobic exercise does stimulate muscle protein synthesis, its primary aim is to reduce skeletal muscle fat infiltration and improve overall muscular system function [62].

Regarding other types of exercise such as whole-body vibrations and multi-component exercises, our study results showed no significant positive effect on lean body mass (including two RCTs on whole-body vibration training and two RCTs on multi-component exercises). This is consistent with a systemic review and meta-analysis conducted by Rubio-Arias et al. which included five studies on whole-body vibration training in postmenopausal women aged 55 to 75 years and found no significant effect on lean body mass [14]. Additionally, a literature review by Bao et al. involving 22 studies on various exercise types (resistance exercise, aerobic exercise, strengthening exercise, multi-component exercise, and vibration exercise) in older adults aged 60 to 86 years did not find a significant difference in overall skeletal muscle mass [63]. It is important to note that the study by Bao et al. focused more specifically on aerobic exercise intervention, resulting in heterogeneity among the included studies due to the varied exercise types and regimens employed [63]. Considering the limitations of our study, which only included 4 RCTs, further research is warranted to validate these findings. Future studies should explore the effects of different exercise modalities and regimens on lean body mass in a more diverse and larger sample population (middle-aged women). Additionally, longer-term interventions and investigations into the underlying mechanisms of exercise-induced changes in lean body mass would provide valuable insights for optimizing exercise interventions in various age groups, especially in middle-aged women.

Another subgroup analysis, conducted by Vikberg et al., focused on the duration of exercise [64]. In their randomized controlled trial, they investigated pre-sarcopenic older adults with an average age of 70 years who underwent 10 weeks of resistance training. The study demonstrated a significant improvement in lean body mass, with an increase of 1.1 kg. Similarly, Liao et al. conducted RCTs involving sarcopenic obese women with an average age of 67.3 years [65]. These participants underwent 12 weeks of resistance band exercise and experienced an increase of 0.79 kg in leg lean mass and 2.19 kg in handgrip strength.

In line with these findings, a meta-analysis conducted by Thomas et al. which included 26 studies involving postmenopausal women aged 50 to 80 years, revealed that 16 weeks of resistance training resulted in an increase in lean body mass [16], similar to the results of our study.

In our study, we performed a subgroup analysis of exercise duration, categorizing it into two groups: ≤ 12 weeks (ranging from 6 to 12 weeks) and > 12 weeks (ranging from 16 to 52 weeks). We observed small to moderate significant increases in lean body mass in menopausal women aged 40 to 60 years. These findings are consistent with a review study by Nascimento et al. which suggested that the sarcopenic population should engage in exercise training for at least 4 weeks or more [66]. Furthermore, Benton et al. recommended that untrained middle-aged women aged 40 to 55, undertaken 8 weeks of resistance training, this regimen is proposed to stimulate muscle protein synthesis, enhance muscle activation, prevent muscle loss, diminish intramuscular fat infiltration, and bolster muscle strength and morphology [58]. Regarding the frequency of exercise training, our subgroup analysis based on two frequencies, namely 20 to 40 min/three sessions per week and 60 to 90 min/three sessions per week, revealed moderate and small effects on lean body mass, respectively. The validated training frequencies were derived from the research studies conducted by Huovinen et al. and Fisher et al. [67, 68]. Huovinen et al. conducted a study involving older women with an average age of 71.9 years [67]. The participants underwent 60 min of exercise training, three sessions per week, for 16 weeks. The study divided exercise frequency and volume into three groups: group 1 performed 20 to 35 min as a low dose of resistance training (RT), group 2 performed 40 min as a moderate dose of RT, and group 3 performed 60 min as a high dose of RT. Each group participated in three sessions per week. All three groups demonstrated significant improvements in muscle mass and muscle strength in response to their respective RT frequencies and volumes [68]. Fisher et al. supported the idea of a minimal dose of exercise for muscle mass and strength improvement, with a focus on major muscle groups [68]. The recommendation is to choose multi-joint exercises that prioritize major muscle groups. Multi-joint exercises include chest press, leg press, and seated row, primarily targeting major muscle groups. These studies emphasize the importance of choosing an appropriate frequency and volume of resistance training for improving muscle mass and strength. Both lower and higher frequencies demonstrated positive responses in terms of muscle mass improvement. However, it is worth noting that the minimal dose of resistance training with a focus on major muscle groups has shown promising results for muscle mass and strength gains.

Handgrip strength is considered the most objective and widely used measure of muscle strength in the upper limb [69]. The meta-analysis conducted in this study has revealed a large effect of exercise training on handgrip strength. In support of our findings, a systematic review by Wang et al. analyzed 23 studies involving older adults with sarcopenia [70]. The studies included multi-component exercise interventions ranging from 9 to 24 weeks in duration. The analysis demonstrated a significant improvement of 2.38 kg in handgrip strength. Moreover, a randomized controlled trial conducted by Toselli et al., participants with an average age of 56.2 years underwent a 24-week program with three sessions per week, has revealed a significant increase of 6.1 kg in handgrip strength [72]. These results align with the overall findings of our study. The consistent results between our meta-analysis and Wang et al.‘s systematic review provide further support for the positive effects of exercise training on handgrip strength in various populations, including sarcopenia older adults. Handgrip strength serves as an important indicator of upper limb muscle strength and can be effectively improved through appropriate exercise interventions.

Knee extension strength is another important muscle measurement frequently used in research, and it was included in our analysis. The results of our study revealed a moderate effect of exercise training on knee extension strength, which is consistent with the findings of a previous review conducted by Lu et al. [71]. Lu et al. conducted a meta-analysis that included 26 studies involving older adults with an average age ranging from 60.6 to 89.5 years [71]. These studies investigated the effects of various types of training, including resistance, endurance, aerobic exercises, and whole-body vibration training, on knee extension strength. The meta-analysis showed significant improvements in knee extension strength with resistance training exercise (1.36 Nm/kg), whole-body vibration training (0.62 Nm/kg), and mixed training (0.62 Nm/kg). Furthermore, Borba-Pinheiro et al. conducted a randomized controlled trial (RCT), participants with an average age of 58.8 years, undergoing 52 weeks of resistance training; there was a significant increase of 15.28 kg in knee extension strength [72]. The improvements in knee extension strength observed in response to exercise training can be attributed to the activation of muscle nerve fibers, increased size of muscle fibers, and enhancement of muscle regeneration. Increased muscle strength can have positive effects on static balance and reduce the risk of falls [73].

This study revealed a low effect of vitamin D supplementation on handgrip strength. This finding is consistent with a meta-analysis conducted by Zhang et al. which included 13 studies involving postmenopausal women over 60 years old [19]. In that meta-analysis, participants were supplemented with varying doses of vitamin D2 (ranging from 2800 IU to 210,000 IU) or vitamin D3 (ranging from 7,000 IU to 100,000 IU) for a duration of 3 to 24 months. The results showed a beneficial improvement of 0.876 kg in handgrip strength. This finding aligns with the results of our study, indicating that vitamin D plays a crucial role in increasing calcium absorption and enhancing muscle tissue repair, leading to improvements in muscle strength [11]. Our study included 4 RCTs that examined different forms and doses of vitamin D supplementation. The interventions ranged from weekly doses of 7,000 IU to 50,000 IU of vitamin D, weekly doses of 40,000 IU of vitamin D2, to a daily dose of 0.5 µg of vitamin D3, administered over a period of 8 to 12 weeks. The heterogeneity in the interventions, including variations in the form and dose of vitamin D, highlights the need for further research to confirm the evidence regarding the effects of vitamin D supplementation on handgrip strength among middle-aged women.

This study found no significant effect of vitamin D supplementation on knee extension strength, which is consistent with the findings of a previous review conducted by Bislev et al. [74]. The systematic review and meta-analysis by Bislev et al. examined older adults with an average age of 65 years who were supplemented with either 10,000 IU of vitamin D2 daily, 400 IU daily, or a single dose of 300,000 IU of vitamin D3 for a duration of 60 months [74]. The results of that review indicated no significant improvements in knee extension strength. Similarly, our study included 3 eligible studies in which participants were supplemented with daily doses of 7,000 IU to 50,000 IU of vitamin D or a weekly dose of 5,000 IU of vitamin D2 for a duration of 8 to 12 weeks. Due to the variations in the forms, dosages, and duration of vitamin D supplementation, the exact beneficial effect of vitamin D on knee extension strength could not be determined. Further trials are needed to determine the optimal dose, form, and duration of vitamin D supplementation for achieving a beneficial effect on knee extension strength among middle-aged women.

This study found no significant improvements in lean body mass with protein supplementation, which is consistent with the findings of a meta-analysis conducted by Guo et al. [75]. Guo et al.‘s meta-analysis included 17 RCTs involving individuals with an age range of 68 to 82 years [75]. In those studies, daily oral supplementation of 0.857 to 7.5 g of leucine, a branched-chain amino acid found in various food sources, was given for durations of 4 to 48 weeks. The meta-analysis showed no beneficial effect of leucine supplementation on lean body mass, which aligns with the results of our study. Leucine is known for its ability to stimulate protein synthesis and promote muscle building and repair [76]. Borack and Volpi have recommended a daily leucine intake of 55 mg/kg to enhance muscle protein synthesis [77]. However, our study included 3 eligible studies that investigated isoflavones with variations in daily oral protein supplementation ranging from 40 mg to 160 mg. This differs from the previous meta-analysis in terms of the specific protein form and dosage used. Additionally, there were variations in the age of participants across studies, making it difficult to directly compare findings between studies. Further trials are needed to determine the optimal dose, forms, and duration of protein supplementation to verify the potential beneficial effects on lean body mass among middle-aged women.

### Limitations

There are several limitations that should be acknowledged in this study. Firstly, the search was limited to articles published in English and Chinese, which may introduce language bias and potentially exclude relevant studies published in other languages. This could limit the comprehensiveness of the literature review. Secondly, a considerable proportion of the included studies lacked specific information about the generation of randomization sequences, blinding of participants and researchers, and registration of the clinical trial. These factors can introduce biases in the random allocation process, deviate from the intended interventions, and affect the reliability of the reported results. Thirdly, a small percentage of participants were aware of their assigned interventions, which may have influenced their performance and introduced performance bias. Additionally, the absence of pre-specific plans or clinical trial registration for analysis in the majority of the included studies may introduce selection bias. Moreover, due to insufficient data and a limited number of RCTs, it was not possible to perform a meta-analysis on the effects of exercise training, vitamin D supplementation, and protein supplementation on physical functional performance in menopausal women aged 40 to 60. Therefore, the study is unable to provide specific recommendations regarding these aspects. Finally, the variations in exercise dose (types, duration, frequency, intensity) across the included studies limited the ability to conduct subgroup analyses and provide detailed recommendations in these specific areas. The available data may not be sufficient to draw conclusive findings in this regard.

### Suggestion

Based on the limitations identified, it is recommended that future research focuses on conducting larger and well-designed RCTs to examine the effects of exercise, vitamin D supplementation, and protein supplementation on muscle health in menopausal women aged 40 to 60. Additionally, systematic reviews with appropriate analytical methods should be performed to determine the optimal dosage and duration of these interventions. It is important to incorporate well-designed RCTs with rigorous randomization, allocation concealment, and blinding of interventions to improve the internal validity of the findings. Pre-specifying analysis plans and registering clinical trials will ensure transparency and minimize selective reporting. Furthermore, considering network meta-analyses can provide valuable insights into the comparative effectiveness of different interventions for preventing sarcopenia in menopausal women.

## Conclusions

Moderate certainty evidence supports exercise training as an effective intervention to improve muscle mass and muscle strength, compared to no exercise, in menopausal women aged 40 to 60. Specifically, resistance training with a frequency of three sessions per week, lasting 20 to 90 min, over a period of at least 6 weeks has been identified as the most beneficial approach. There is moderate to low certainty evidence suggesting that vitamin D supplementation may offer potential benefits for strength in smaller muscle groups. Additionally, while low certainty evidence points to protein might be used as a potential non-pharmacological intervention for sarcopenia prevention, further research is needed to determine the optimal dosage and forms. Overall, cultivating a regular exercise habit is crucial for preventing sarcopenia and improving quality of life in menopausal women. It is recommended to implement evidence-based health promotion policies to raise awareness and knowledge about the risks of sarcopenia during menopausal transition and to encourage preventive measures.

## Relevance to clinical practice

The clinical implications of our study suggest that menopausal women aged 40 to 60 should maintain regular exercise habits and choose exercises that suit their lifestyles. Specifically, a training duration of 6 weeks or more, three sessions per week, and 20 to 90 min per session have shown to be effective. Beginners can start by incorporating exercise into their daily routine without being constrained by time or fitness venues. To increase physical activity, women can take stairs instead of elevators and use public transit. Resistance exercises, such as leg press, seated rows with elastic bands, leg extensions, seated leg curls, and dumbbell bench presses, can be performed at home with sports equipment. Regarding vitamin D supplementation, caution is advised due to variations in dosage and forms. Non-pharmacological strategies to prevent sarcopenia and improve quality of life during menopause should be emphasized. Furthermore, public health nurses act as key intervention providers, are important to deliver non-pharmacological intervention to prevent sarcopenia (loss of muscle mass, muscle strength and physical performance) in this population. Exercise and nutrition education programs for the prevention of sarcopenia will be strategic to improve the quality of life in women during menopausal transition.

### Electronic supplementary material

Below is the link to the electronic supplementary material.

Supplementary Material 1

## Data Availability

The data that support the findings of this study are available from the corresponding author upon reasonable request.

## Abbreviations

IU (international unit): A unit of measurement for vitamins

## Declarations

**Acknowlegements**:
